# Valorization of Brewery Waste through Polyhydroxyalkanoates Production Supported by a Metabolic Specialized Microbiome

**DOI:** 10.3390/life12091347

**Published:** 2022-08-30

**Authors:** Mónica Carvalheira, Catarina L. Amorim, Ana Catarina Oliveira, Eliana C. Guarda, Eunice Costa, Margarida Ribau Teixeira, Paula M. L. Castro, Anouk F. Duque, Maria A. M. Reis

**Affiliations:** 1 Associate Laboratory i4HB—Institute for Health and Bioeconomy, NOVA School of Science and Technology, NOVA University Lisbon, 2819-516 Caparica, Portugal; 2 UCIBIO—Applied Molecular Biosciences Unit, Department of Chemistry, NOVA School of Science and Technology, NOVA University Lisbon, 2819-516 Caparica, Portugal; 3 Universidade Católica Portuguesa, CBQF—Centro de Biotecnologia e Química Fina—Laboratório Associado, Escola Superior de Biotecnologia, Rua Diogo Botelho 1327, 4169-005 Porto, Portugal; 4 CENSE—Center for Environmental and Sustainability Research & CHANGE—Global Change and Sustainability Institute, Faculty of Sciences and Technology, University of Algarve, Campus de Gambelas, 8005-139 Faro, Portugal

**Keywords:** biopolymers, circular economy, waste valorization, microbial community

## Abstract

**Simple Summary:**

Raw brewers’ spent grain, a by-product of beer production, is produced at a large scale and is usually used as animal feed or is landfilled. However, its composition shows that this feedstock has the potential for other applications, such as bioplastics production (e.g., polyhydroxyalkanoates). In this way, the aim of this work was to assess the use of raw brewers’ spent grain for polyhydroxyalkanoates production, adding new value to this feedstock. The results confirm the potential of raw brewers’ spent grain to produce polyhydroxyalkanoates, as the population was enriched in the microorganisms able to accumulate these biopolymers. These results will contribute to society’s knowledge and competence via the development of a treatment process for brewery waste of both environmental (productive waste treatment) and economic interest (production of biopolymers), which will certainly attract its application to the brewery industry worldwide.

**Abstract:**

Raw brewers’ spent grain (BSG), a by-product of beer production and produced at a large scale, presents a composition that has been shown to have potential as feedstock for several biological processes, such as polyhydroxyalkanoates (PHAs) production. Although the high interest in the PHA production from waste, the bioconversion of BSG into PHA using microbial mixed cultures (MMC) has not yet been explored. This study explored the feasibility to produce PHA from BSG through the enrichment of a mixed microbial culture in PHA-storing organisms. The increase in organic loading rate (OLR) was shown to have only a slight influence on the process performance, although a high selectivity in PHA-storing microorganisms accumulation was reached. The culture was enriched on various PHA-storing microorganisms, such as bacteria belonging to the *Meganema*, *Carnobacterium*, *Leucobacter*, and *Paracocccus* genera. The enrichment process led to specialization of the microbiome, but the high diversity in PHA-storing microorganisms could have contributed to the process stability and efficiency, allowing for achieving a maximum PHA content of 35.2 ± 5.5 wt.% (VSS basis) and a yield of 0.61 ± 0.09 Cmmol_PHA_/Cmmol_VFA_ in the accumulation assays. Overall, the production of PHA from fermented BSG is a feasible process confirming the valorization potential of the feedstock through the production of added-value products.

## 1. Introduction

During the last few years, population growth has led to an increase in industrialization in order to ensure population needs, causing an increase in the quantity and variety of waste generated, as well as an increase in the production and utilization of petrochemical products, namely plastics [1,2,3]. Consequently, the interest in renewable resources has been increasing due to the rising awareness regarding the use of more environmentally friendly products, such as polyhydroxyalkanoates (PHAs), which are biodegradable, biocompatible polyesters that present properties that are similar to conventional plastics [4]. These bioplastics, which can be produced from wastes, have numerous potential applications, thus contributing to a circular economy and to the Sustainable Development Goals [3,5,6,7]. PHAs can be produced using microbial mixed cultures (MMC) and wastes in a three-stage process: (1) acidogenic fermentation, where organic matter is converted into volatile fatty acids (VFAs), the precursors of PHA; (2) selection of MMC, where the microbial culture is subjected to a selective pressure (e.g., feast and famine regime), enriching the culture in PHA accumulating organisms; and (3) PHA production, where the VFAs produced in the first stage are used to feed the selected MMC, aiming at the culture’s maximum PHA accumulation [3,8,9]. When compared with the production by pure cultures, the production of PHA by MMC using waste presents economic advantages, namely low production costs, as it allows for the use of open systems and low-cost substrates, which will contribute to a reduction in PHA production costs [5,7,10]. Although there are advantages, the production of PHA by MMC still presents some challenges, namely, a consistent PHA composition assuring constant properties [9], relatively low productivities when wastes are used as feedstocks, and low production costs when compared with the production of conventional plastics [11].

Brewers’ spent grain (BSG) is a by-product of beer production, obtained from wort filtration. It represents about 85% of the total by-products generated during beer production [12,13,14]. BSG is a lignocellulosic material composed of fibers (ca. 70% of cellulose, hemicellulose and lignin), proteins (ca. 30%), minerals, and lipids [13,14,15,16]. However, the chemical composition of this residue depends on the variety of barley used, the harvest time and cultivation, and the malting and mashing conditions [15]. The availability of this kind of residue and its reuse/valorization is still limited to animal feed or it is landfilled [12,14]. Nevertheless, because of its composition, BSG is a potential feedstock for several applications, namely for polymers production [16]. Currently, BSG has been used to produce PHAs using single strains (*Burkholderia cepacia*, *Bacillus cereus*, and *Cupriavidus necator*), achieving a PHA yield of up to 23 mg PHA/g BSG using *B. cepacia*, showing the potential application of BSG as a feedstock for added-value product production [17,18,19,20]. The PHA production by MMC can be more economically advantageous over pure/single culture production; however, to the best of our knowledge, this has not yet been reported. The composition of BSG (lignocellulosic and recalcitrant materials content), as well as the solid-state form of this raw waste, present a challenge for its use in biological processes, as it is usually necessary for a pre-treatment to increase the process performance. However, this challenge can be overcome when MMCs are used, as the BSG can be fermented in the solid state, producing a VFA-rich stream [12], and then fed to MMC for PHA production, presenting an advantage over single cultures that usually use the BSG hydrolyzed or supplemented with other carbon sources to increase the PHA productivity [17,18,19,20]. Moreover, the use of fermented BSG by MMC allows for producing PHA with different compositions, depending on the VFA profile, which represents an advantage for PHA application. Therefore, in this work, the feasibility of producing PHA using fermented BSG as a feedstock was assessed at distinct OLRs while monitoring the response of the reactor microbiome towards these process variations. The knowledge on the dominant population under specific process conditions can help in devising the microbial consortia required for a robust and efficient BSG valorization approach.

## 2. Materials and Methods

### 2.1. Feedstock Characterization

**Figure 1:**
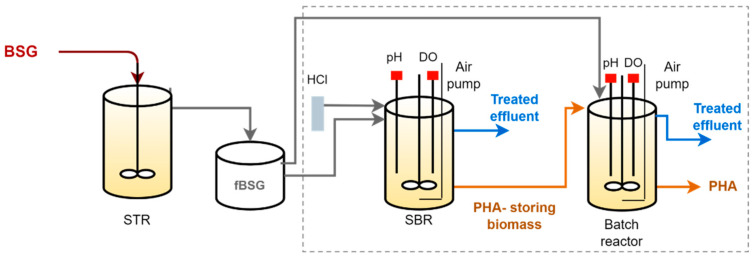
Three-stage PHA production set-up: this study focused on MMC enrichment (SBR) and PHA production (batch reactor) (highlighted by the dashed line); the production of the fBSG was published by Teixeira et al. [12].

Fermented brewers’ spent grain (fBSG) produced in a previous work [12] was used as the feedstock (Figure 1). Briefly, the raw BSG was fermented using a fed batch stirred tank reactor operated under an organic loading rate (OLR) of between 4.3–16 g TS/(L·d). The fBSG was composed of a mixture (% C mol basis) of: 29.4 ± 3.1% acetic acid, 36.6 ± 5.6% propionic acid, 0.9 ± 0.2% isobutyric acid, 22.2 ± 6.0 % butyric acid, 2.1 ± 0.4% isovaleric acid, and 8.6 ± 1.5% valeric acid. This composition corresponded to an estimated polymer composition of 53:47 C mol% HB:HV, considering acetic, isobutyric, and butyric acids as HB precursors, and propionic, isovaleric, and valeric acids as HV precursors. Moreover, the fBSG presented a C:N:P ratio of 100:8:2 Cmol:Nmol:Pmol, eliminating the need for nutrient supplementation in the culture selection stage. Sugars, namely xylose, arabinose, glucose, and maltose, were not detected in the fBSG composition. The VFA content over the soluble chemical oxygen demand (COD) corresponded to about 81%, indicating the presence of other carbon sources, such as other fermentable sugars (not detected by the HPLC method used, as described in Section 2.3) and/or proteins, in the fBSG.

### 2.2. Experimental Setup

#### 2.2.1. MMC Enrichment in PHA-Storing Organisms

A 2 L working volume sequencing batch reactor (SBR), inoculated with aerobic activated sludge from a municipal wastewater treatment plant (Almada, Portugal), was used for culture selection (Figure 1). The SBR was operated under a feast and famine regime for 80 days. Each SBR cycle length was of 12 h, comprising five distinct periods: filling (5 min), aeration (feast and famine) (675 min), purge (1 min), settling (30 min), and withdrawal of the exhausted effluent (9 min). The hydraulic retention time (HRT) and sludge retention time (SRT) were controlled at 1 day and 4 days, respectively. The SBR was operated at room temperature and the pH was set at 8.00 ± 0.5 through the automatic addition of 0.5 M HCl. Air was supplied with a flow between 1.5 and 2 L/min, and stirring was maintained at 350 rpm. The organic loading rate (OLR) increased from 19.2 ± 3.1 to 48.6 ± 7.0 Cmmol/(L·d) with a C:N:P ratio, on average, of 100:8:2 Cmol:Nmol:Pmol. The SBR was fed with the fBSG that was diluted with tap water according to the OLR applied (Table 1) and was supplemented with allythiourea (10 mg/L) to avoid nitrification. Dissolved oxygen (DO) and pH data were acquired continuously, which allowed for online monitoring of the feast phase length. The SBR performance was monitored along the cycles where the PHA, volatile fatty acids (VFA), nutrients, and total and volatile suspended solid (TSS and VSS) contents were analyzed. In addition, biomass samples were taken for Nile blue staining and DNA analysis.

#### 2.2.2. PHA Accumulation

From day 64 onwards, PHA accumulation assays were performed using the enriched SBR culture and were fed with fBSG in pulse wise mode controlled by the DO level (Figure 1). Prior to the assays, the pH of the feed was adjusted to 7.5 by adding NaOH to avoid a pH decrease after the pulse feeding. The reactor was operated with no pH control and at room temperature. The DO (2–7 mgO_2_/L) and pH (8.0–8.8) were continuously monitored. When the DO increased, a new pulse was fed to the reactor. This procedure was repeated until the culture reached its maximum capacity. The assay performances were monitored along the pulses for the same parameters as for the SBR operation.

### 2.3. Analytical Methods

TSS and VSS were determined according standard methods [21]. The VFAs (acetic, propionic, isobutyric, butyric, isovaleric, and valeric acids) concentration was determined by high-performance liquid chromatography (HPLC) using a VWR Hitachi Chromaster chromatographer with an RI detector, a Biorad Aminex HPX-87H column (300 mm × 7.8 mm), and a Biorad pre-column (125-0129, 30 mm × 4.6 mm). The analysis was performed using sulfuric acid 0.01 N as the eluent at a 0.5 mL/min flow rate and 30 °C. The VFAs concentration was calculated through calibration curves in the range of 3.9 to 1000 mg/L. The nutrients (ammonium and phosphorus) concentrations were determined using a colorimetric method implemented in a continuous flow analyzer (Skalar San ++, Skalar Analytical, The Netherlands).

PHAs quantification was performed according to Duque et al. [9]. Briefly, the biomass was lyophilized and weighted (3–8 mg) and 1 mL of acidic methanol (20% sulfuric acid) and 1 mL of chloroform were added. The samples were digested at 100 °C for 3.5 h. After digestion, the organic phase that contained the PHAs was extracted and injected (2 μL) in a gas chromatograph with a Flame Ionization Detector (Gas Chromatograph 430-GC, Bruker), equipped with a Restek column (60 m, 0.53 mm internal diameter, 1 μm film thickness, Crossbond, Stabilwax). The carrier gas was helium, at a flow rate of 1 mL/min. The standard used for polyhydroxybutyrate (PHB) and polyhydroxyvalerate (PHV) quantification was a copolymer of P(HB/HV) (88%/12% mol) (Sigma). Heptadecane was used as the internal standard (concentration ca. 1 g/L).

Nile blue staining was used to identify the intracellular PHA granules, as described by Bengtsson et al. [22]. Briefly, 20 μL of Nile blue were added to 1.5 mL of the sample in an eppendorf. The sample was incubated at 55 °C for 10 min. After that time, the sample was observed using a microscope (Olympus BX51 epifluorescence) under fluorescent light, which allowed for observing the accumulated PHA granules.

The microbial culture was identified through 16S rRNA gene amplicon sequencing targeting the bacterial variable regions V1–V3 using Illumina technology. The DNA extraction and bioinformatic processing was carried out by DNASense, Aalborg, Denmark.

### 2.4. Calculations

The specific substrate uptake rate (-qS in Cmmol_VFA_/(Cmmol_X_·h) and in (gCOD_VFA_/(gCOD_X_·h)) and PHA storage rate (qPHA in Cmmol_PHA_/(Cmmol_X_·h) and in gCOD_PHA_/(gCOD_X_·h)) were determined by linear regression of the substrate, PHA, and active biomass (X) specific concentrations plotted over time, respectively. The PHA content (% gPHA/gVSS) was determined as a percentage of VSS on the mass basis, where PHA corresponds to the sum of PHB and PHV, and VSS corresponds to the sum of the active biomass (X) and PHA. The PHA storage yield and growth yield per substrate consumed (Y_PHA/VFA_ in Cmmol_PHA_/Cmmol_VFA_ and in gCOD_PHA_/gCOD_VFA_; Y_X/VFA_ in Cmmol_X_/Cmmol_VFA_ and in gCOD_X_/gCOD_VFA_) were determined through the division of the specific PHA storage rate and growth rate by the specific substrate uptake rate, respectively. The growth yield during the famine phase (Y_X/PHA_ in Cmmol_X_/Cmmol_PHA_ and in gCOD_X_/gCOD_PHA_) was determined by dividing the growth rate by the specific PHA consumption rate. The feast/famine ratio (F/f, h/h) was calculated by dividing the length of the feast by the length of the famine phase in the SBR cycle. The biomass productivity was determined as described in Matos et al. [23]. The PHA productivity was determined by taking into account the PHA produced by unit of time. Standard deviations were determined using standard deviation propagation formulae.

## 3. Results and Discussion

### 3.1. Culture Enrichment on PHA-Storing Organisms

The SBR was operated for 80 days under a regime of feast and famine to select a culture rich in PHA-storing organisms. The F/f ratio is generally used as an indicator of an efficient culture selection, as an F/f ratio below 0.2 h/h has been proven to ensure an effective selective pressure for PHA-storing organisms [9,22]. Under the initial OLR of 19.2 ± 3.1 Cmmol/(L·d) and after 8 days of operation, a F/f ratio lower than 0.2 h/h was achieved (0.07 ± 0.01 h/h) and maintained during the remaining operation period (0.09 ± 0.03 h/h), except for a short period after the increase of OLR to 36.8 ± 2.8 Cmmol/(L·d), where the F/f varied between 0.20 and 0.32 h/h.

The step wise increase in the OLR (from 19.2 ± 3.1 to 48.6 ± 7.0 Cmmol/(L·d)) aimed to acclimatize the culture to the feedstock and to increase the process productivity. Indeed, the biomass concentration and, consequently, the biomass productivity increased with the increase in OLR (2.9 times), achieving a maximum of 0.56 ± 0.02 g_X_/(L·d) at an OLR of 48.6 Cmmol/(L·d) (Table 1). This increase in the biomass concentration with the OLR was also observed in other studies [24,25]. The biomass productivity was shown to be an important parameter in the PHA production process, as the PHA productivity can be affected by the amount of biomass available for its production, as a high selected biomass concentration could lead to a higher amount of PHA produced [26].

**Figure 2:**
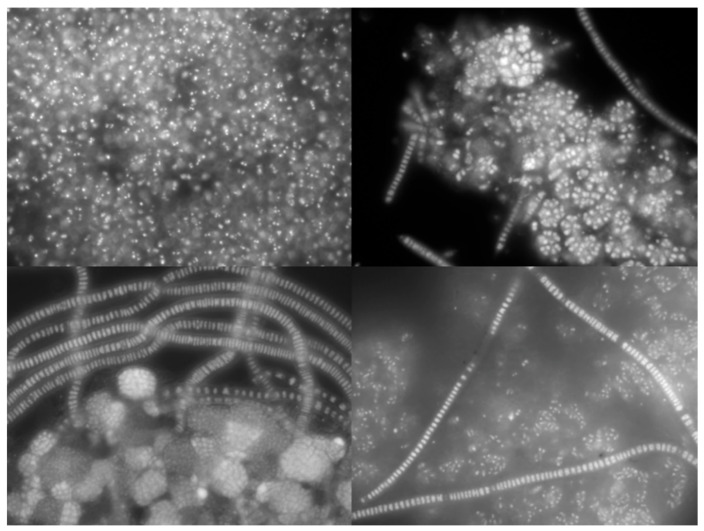
Nile blue images obtained from the observation of the reactor biomass samples of the selected culture collected under the highest OLR applied. The white granules correspond to PHA granules.

Overall, the increase in the OLR did not seem to influence the reactor performance as the kinetic and stoichiometric parameters did not vary substantially (Table 1), showing the robustness of the MMC selected and the process. The substrate consumption and PHA storage rates were similar for all the conditions tested, indicating that the kinetic parameters seemed to be independent of the OLR range applied. The substrate consumption rate obtained for the highest OLR applied (0.43 ± 0.05 Cmmol_VFA_/(Cmmol_X_·h); 0.51 ± 0.07 gCOD_VFA_/(gCOD_X_·h)) was in the same range as the ones obtained for other fermented streams with similar VFA compositions and operated at a similar OLR (0.34–0.50 Cmmol_VFA_/(Cmmol_X_·h) [9,27]). However, the obtained substrate consumption rates were lower than those reported in the studies that used synthetic mixtures of VFAs (0.63–0.92 Cmmol_VFA_/(Cmmol_X_·h)), but that applied higher OLRs [26,28]. Regarding the consumption of specific acids, under the highest OLR applied, the culture showed a preference for propionic and butyric acids (0.159 ± 0.023 Cmmol_VFA_/(Cmmol_X_·h) and 0.155 ± 0.012 Cmmol_VFA_/(Cmmol_X_·h), respectively), followed by acetic and valeric acids (0.091 ± 0.007 Cmmol_VFA_/(Cmmol_X_·h) and 0.074 ± 0.007 Cmmol_VFA_/(Cmmol_X_·h), respectively) (data not shown). This preference by butyric acid was also observed in other studies [23,28,29] and could be related to the fact that butyric acid uptake is energetically more efficient than those of the other acids [23]. A PHA storage rate of 0.34 ± 0.04 Cmmol_PHA_/(Cmmol_X_·h) (0.39 ± 0.04 gCOD_PHA_/(gCOD_X_·h)) was attained at the highest OLR of 48.6 ± 7.0 Cmmol/(L·d), which is in accordance with the ones of other studies using fermented streams (up to 0.4 Cmmol_VFA_/(Cmmol_X_·h) [8,9,27]; up to 0.48 gCOD_VFA_/(gCOD_X_·h) [27,30]). The PHA storage yield showed the good capacity of the MMC to accumulate PHA (Figure 2), obtaining a maximum content of 24.2 ± 3.8 wt.% under the highest OLR applied, which corresponds to an average PHA yield of 0.79 ± 0.04 Cmmol_PHA_/Cmmol_VFA_ (0.76 ± 0.04 gCOD_PHA_/gCOD_VFA_). This yield was similar or even higher to those obtained in other studies (0.66–0.90 Cmmol_PHA_/Cmmol_VFA_ using fermented cheese whey and sugar molasses [8,9,24,27]; up to 0.43 Cmmol_PHA_/Cmmol_VFA_, using brewery wastewater [31]; up to 0.57 gCOD_PHA_/gCOD_VFA_, using a synthetic mixture of organic acids [32]; up to 0.56 gCOD_PHA_/gCOD_VFA_, using fermented olive oil mill wastewater [30]). The polymer composition produced under each OLR was similar, varying between 55:45 wt.% HB:HV and 46:54 wt.% HB:HV for the lowest and highest OLRs, respectively, and was similar to the estimated polymer composition based on the HB and HV precursors content on the fermented feedstock (53:47 wt.% HB:HV) (Table 1).

The high PHA storage yield compared with the growth yield during the feast (0.04–0.08 Cmmol_X_/Cmmol_VFA_) showed the dominance of PHA storage processes over biomass growth in terms of carbon uptake, which confirms efficient MMC selection by the selective pressure applied (short feast and long famine periods). The main biomass growth occurred during the famine phase, as a high growth yield was obtained (ca. 4 to 6 times higher than in the feast phase) (Table 1).

### 3.2. Assessment of PHA Production

**Figure 3:**
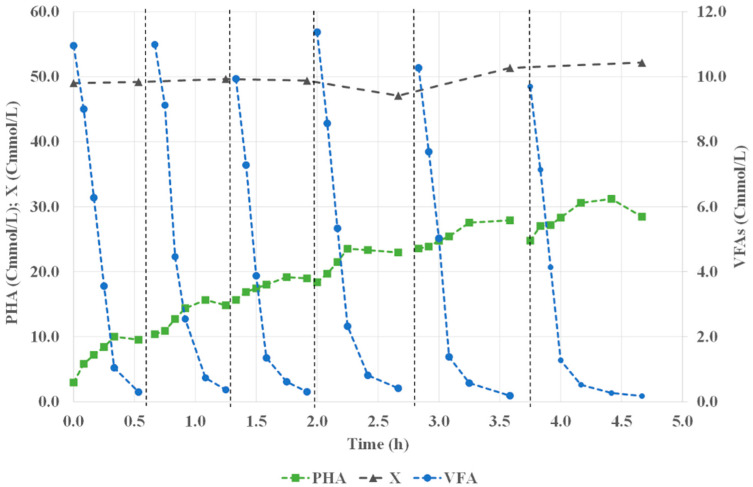
Typical profile of the accumulation assays performed with the culture selected at OLR 48.6 Cmmol/(L·d). The six pulse periods are limited by the vertical dashed lines.

The assessment of the PHA production capacity of the selected culture was performed using biomass collected from the SBR operated under the highest OLR (48.6 ± 7.0 Cmmol/(L·d)) at the pseudo-steady state. Figure 3 presents the typical PHA, VFA, and active biomass concentration profiles observed during the accumulation assays. In each pulse feeding, it was possible to observe the consumption of the VFAs and the subsequent increase in the PHA concentration along the assay. On average, a specific substrate consumption rate of 0.58 ± 0.09 Cmmol_VFA_/(Cmmol_X_·h) was obtained in the PHA accumulation assays, which was higher than the one obtained during the culture selection stage, but comparable with the ones of other studies (0.42–0.54 Cmmol_VFA_/(Cmmol_X_·h)) [8,9,24,27,33].

The maximum PHA content obtained in the accumulation assays was 35.2 ± 5.5 wt.% (VSS basis), which was lower than those obtained in other studies with different feedstocks (49–81 wt.%) [9,32,33]. The lower PHA content could be related to the lower amount of carbon fed to the culture during the assays when compared with the organic load applied in the other studies. Nevertheless, this result showed the ability to produce PHAs from fBSG, although an optimization of the process is still needed to improve the process performance and productivity. The PHA yields obtained in the accumulation assays, considering the two first pulses of the three assays, were lower than the ones obtained in the selection stage (varied between 0.52 and 0.72 Cmmol_PHA_/Cmmol_VFA_ with an average yield of 0.61 ± 0.09 Cmmol_PHA_/Cmmol_VFA_), probably due to the PHA consumption observed during the pulses (data not shown), which led to a decrease in the maximum PHA storage capacity of the selected culture. The increase in the cell content in PHAs probably led to a reduction in PHA production and, consequently, the PHA yield for the last pulses decreased to 0.32 ± 0.06 Cmmol_PHA_/Cmmol_VFA_. Nonetheless, the PHA yield was comparable to those reported in previous studies applying a similar organic load (up to 0.70 Cmmol_PHA_/Cmmol_VFA_) [9,24,27]. A maximum PHA storage rate of 0.35 ± 0.09 Cmol_PHA_/(Cmol_X_·h) (0.40 ± 0.10 gCOD_PHA_/(gCOD_X_·h)) was obtained, which was similar to those reported in previous studies [8,9,27,33]. Regarding the PHA monomeric composition, an average ratio HB:HV of 50:50 Cmol % was obtained, corresponding approximately to the PHA composition predicted from the fermented BSG composition (53:47 HB:HV Cmol%) (data not shown).

The average PHA productivity, considering the maximum PHA produced during the accumulation assays was of 4.12 ± 0.89 g_PHA_/(L·d), which was within the range of PHA productivities reported in previous studies (1.50–8.90 g_PHA_/(L·d)) [8,9,30,32]. Although the process is still not optimized, a preliminary assessment of the process efficiency estimated that the production of 1 kg of PHA required 13.9 kg of dry BSG, resulting in a global yield of 0.07 kg_PHA_/kg_dry BSG_ (taking into account the parameters obtained in the present study as well as in Teixeira’s study [12]).

### 3.3. Microbiome Composition Dynamics

#### 3.3.1. Richness and Diversity

To figure out the effect of the OLR applied on the microbiome, high-throughput 16S rRNA gene sequencing was performed on biomass samples collected over the reactor operation.

Alpha-diversity analysis was used to estimate the diversity and richness of the bacterial communities within biomass over time and is summarized in Table 2. The inoculum presented the highest Shannon index (3.4), indicating that the microbiome on that day was highly diverse. During the reactor operation, a loss in microbiome diversity was observed, reaching the lowest level at day 63, where the Shannon index was 2.0. This was probably due to the enrichment process occurring during the reactor operation, which led to a specialization of the bacterial community. The richness of the bacterial community each day was estimated using the Chao-1 index. The biomass on day 0 presented a higher bacterial richness, which was lost during the reactor operation. In fact, previous studies also reported a reduction in microbial diversity (such as Shannon diversity) when higher OLRs were applied [34,35].

**Figure 4:**
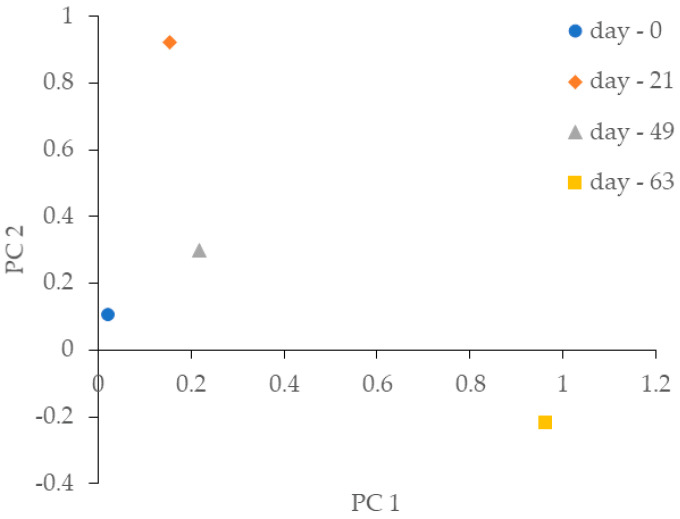
Principal component analysis of the 16S rRNA gene sequencing data at the OTU level from days 0, 21, 49, and 63, where different OLRs were applied. PC1 and PC2 explain 57.6% and 26.9% of the total variance, respectively.

The similarity between the biomass microbiome over time was explored by principal component analysis (PCA) based on operational taxonomic units (OTUs) abundance (Figure 4). The PCA plot shows that samples presented a dispersed distribution with a clear separation between bacterial communities from day 0 and day 63. Feeding with the fBSG and the OLR applied played a crucial role in shaping the biofilm microbiome. Over time, the OLR could have created selective pressure on the bacterial community to regulate metabolic pathways for more efficient PHAs production. Previous studies have shown that the OLR is a selective parameter of microbial populations, influencing the biomass microbial composition profile and consequently the production of PHAs [36].

#### 3.3.2. Taxonomic Composition

**Figure 5:**
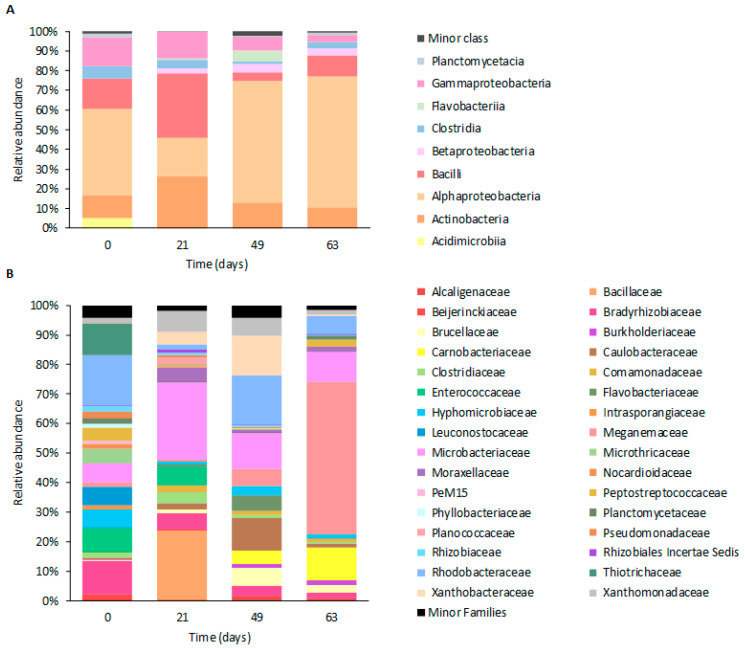
Taxa relative abundance at class (**A**) and family (**B**) levels on the different reactor operational days. Minor classes and minor families include classes and families for which the relative abundances are lower than 1% for all of the sampling days.

Throughout the operation, the microbiome composition of the reactor biomass changed gradually. The reactor inoculum was characterized by the predominance of microorganisms belonging to the *Alphaproteobacteria* class (up to 43% of the classified reads in that day), followed by microorganisms belonging to the *Bacilli* (ca 15%), *Gammaproteobacteria* (ca 14%), and *Actinobacteria* (ca 11%) classes (Figure 5A). At the family level, the most abundant families were *Rhodobacteraceae* (ca 16%), *Bradyrhizobiaceae* (ca 11%), *Thiotrichaceae* (10%), and *Enterococcaceae* (ca 8%) (Figure 5B).

After a period of 21 days of being fed with fBSG at an OLR of 19.2 ± 3.1 Cmmol/(L·d), the microbiome composition shifted. Bacteria belonging to the *Alphaproteobacteria* class (ca 19%) lost its predominance within the biomass and the taxa of the class *Bacilli* (ca 33%) became the most predominant. At the family level, bacterial communities belonging to the *Microbacteriaceae* (ca 26%) and *Bacillaceae* (ca 24%) families prevailed over the remaining families.

On day 22, an increase in the OLR to 36.8 ± 2.8 49 Cmmol/(L·d) was achieved and maintained for 27 days, changing the biomass microbiome composition within the reactor. A reduction in the number of microorganisms affiliated to the *Gammaproteobacteria* (ca 7%) and *Actinobacteria* (ca 13%) classes was observed, while the ones affiliated to the *Alphaproteobacteria* (ca 61%) class increased (Figure 5A). Consequently, on day 49, members of the *Caulobacteraceae* (ca 11%), *Xanthobacteraceae* (ca 13%), and *Rhodobacteraceae* (ca 17%) families were on the top four most abundant bacteria families within the biomass, together with members of the *Microbacteriaceae* family (ca 12%).

From days 50 to 63, the reactor was operated at an OLR of 48.6 ± 7.0 Cmmol/(L·d), which led to the microbiome composition restructuration. On day 63 of operation, nearly half of the biomass bacterial community was composed of microorganisms belonging to the *Meganemaceae* class (ca. 51% of the total relative abundance of the bacterium at the class level) (Figure 5B). The following largest taxonomic groups were the *Microbacteriaceae* (ca 10%) and *Carnobacteriaceae* (ca 11%) families, but accounted for a substantially lower proportion of bacteria-assigned reads. This suggests that the stepwise increase in OLR shifted the microbiome toward a selectively enriched family, which probably influenced the positive performance of the biomass productivity.

#### 3.3.3. Core Bacteriome

**Figure 6:**
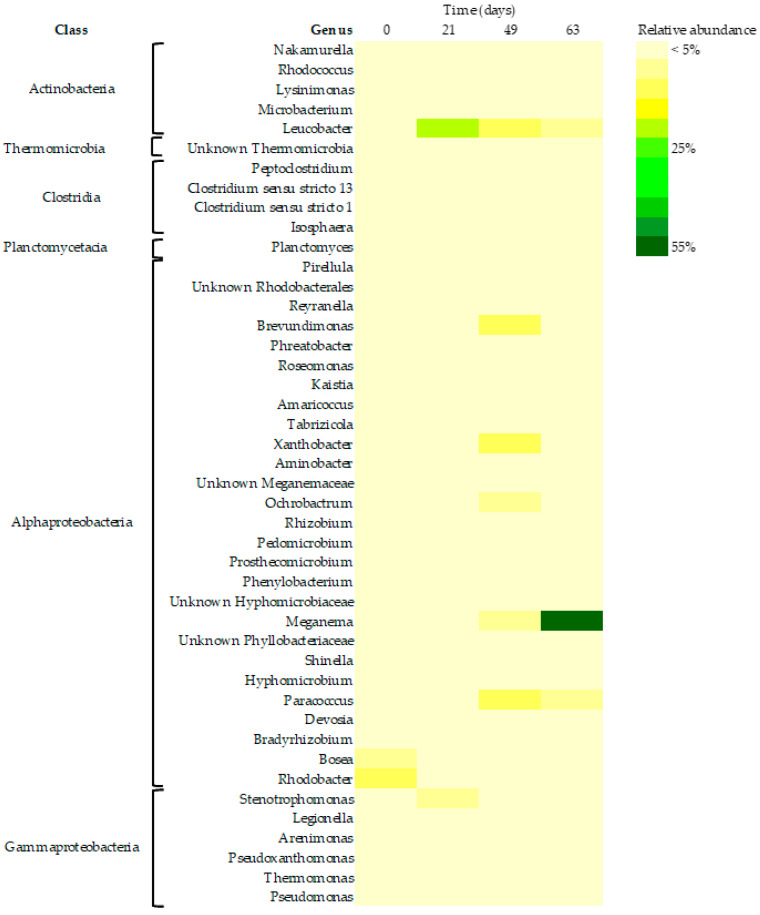
Heatmap of the core microbiome taxa with relative abundance >0.1% across reactor operation. The genus or the lowest assigned taxonomic level of each taxon is presented in the heat map.

A detailed analysis of the microbiome revealed the existence of a core community, composed of 44 distinct bacterial taxa with relative abundances greater than 0.1%, that was found to be present in all sampling days (Figure 6). The core bacteriome members were identified as different members of the *Proteobacteria* (33 taxa), *Actinobacteria* (5 taxa), *Firmicutes* (4 taxa), *Planctomycetes* (1 taxon), and *Chloroflexi* (1 taxon) phyla. About 75% of the bacteria taxa belonged to the Proteobacteria phylum. Previous studies have found that putative PHA-accumulating bacteria in the biomass are mainly represented by members of the *Alphaproteobacteria* and *Gammaproteobacteria* classes, even when the reactor operational conditions are different [37]. In fact, within *Proteobacteria*, the bacterial class *Alphaproteobacteria* harbored different taxa such as *Paracoccus*, *Amaricoccus, Leucobacter, Rhizobium,* or *Meganema,* which are well recognized for their PHA accumulation capabilities [8,35,37,38,39]. All of these microorganisms were present in the inoculum used for the start-up of the reactor, and over reactor operation, due to the enrichment process, the relative abundance of the members from the *Paracoccus, Leucobacter,* and *Meganema* genera increased. Nevertheless, most of the taxa belonging to the core microbiome presented a low (< 5%) but consistent relative abundance over the reactor operation. Core microorganisms have been described as the smallest but functionally indispensable components of the total microbiome, maintaining the ecological stability of the process [34].

**Figure 7:**
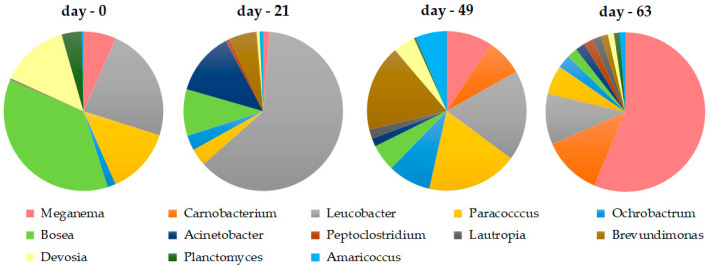
Distribution of the taxa, at a genus level, with a relative abundance higher than 1% on day 63, and their dynamics on the previous days.

The highest biomass productivity was achieved during the last stage of reactor operation when an OLR of 48.6 ± 7.0 Cmmol/(L·d) was applied. Thus, in order to have deeper knowledge about the enriched microbial populations that allowed for a greater production of PHA, pie-charts were elaborated taking into account the taxa with the relative abundance greater than 1% on day 63 (Figure 7).

On day 63, the taxa identified at a relative abundance higher than 1% included well known PHA-storing microorganisms (e.g., *Meganema*, *Amaricoccus*, and *Paracoccus* genera). The reactor biomass was dominated by the bacterial genus *Meganema*, which accounted for ca 51.2% of the total microbiome on that day. The bacteria from this genus has been reported as filamentous bacteria with PHA accumulating abilities. This characteristic made them strong competitors in the system due to their capacity for a high substrate uptake rate in low dissolved oxygen conditions [35]. As such, the proliferation of members of the genus *Meganema* within the system could have limited the proliferation of other bacteria during the enrichment process, which could explain its high relative abundance and also the decrease in diversity indexes. Nevertheless, although at a low relative abundance, bacteria belonging to the *Carnobacterium* (ca 10.9%), *Leucobacter* (ca 9.4%), and *Paracoccus* (ca 5.3%) genera are also well represented in the biomass microbiome on day 63. These three genera are often reported as the main microbial groups responsible for PHA production in MMC systems [8,37,40,41,42,43]. Within the remaining genera present in the biomass microbiome at relative abundances between 1–2.5%, there are also other genera that are reported in the literature as having metabolic constitution enzymes capable of synthesizing PHAs, namely bacteria from the genus *Amaricoccus*, *Bosea*, *Rhodobacter,* or *Peptoclostridium* [8,37,44,45]. However, these genera seemed to be not so well adapted to the operational conditions imposed and thus were not able to thrive over the well-established PHA-storing microorganisms. This is especially evident for the genera *Rhodobacter* and *Bosea*, whose relative abundances decreased over the reactor operation.

Although the highest OLR seemed to favor the proliferation of the *Meganema* genus, which became predominant within the biomass microbiome, other PHA-storing microbes were also able to thrive in such conditions, although likely at a slower rate. This richness in PHA-storing microorganisms is one of the main advantages in MMC as the presence of various microorganisms with overlapping metabolic functions within the same niche allowed for the flexibility and stability of the process.

## 4. Conclusions

The production of PHA by MMC using fBSG was successfully accomplished, highlighting the potential of using solid BSG as feedstock for the production of added value compounds, and at the same time contributing to the environmental sustainability and circular economy strategy in the agro-food sector. The stepwise increase in the OLR only slightly affected the SBR performance, allowing for enrichment of the MMC in PHA-storing organisms. The selective pressure, induced by the applied OLRs, influenced the microbiome composition, enriching it in PHA-storing bacteria. At the highest OLR, members of the genus *Meganema* dominated the biomass microbiome, but other PHA-storing microorganisms such as *Carnobacterium, Leucobacter* and *Paracocccus* were also present in the microbiome.

The diversity in PHA-storing microorganisms in the enriched biomass and the predominance of such microorganisms within the overall reactor microbiome could have contributed to the maximization of the process’s yield and productivity.

## Figures and Tables

**Table 1:** Performance of the MMC selected in the SBR in the feast phase at the pseudo-steady state of each OLR.

Operational Days	0–21	22–49	50–63
**OLR**			
(Cmmol/(L·d))	19.2 ± 3.1	36.8 ± 2.8	48.6 ± 7.0
(gCOD/(L·d))	0.7 ± 0.1	1.3 ± 0.1	1.8 ± 0.3
**Biomass productivity** (g_X_/(L·d))	0.20 ± 0.03	0.35 ± 0.06	0.56 ± 0.02
**-qS** (Cmmol_VFA_/(Cmmol_X_·h))	0.43 ± 0.11	0.42 ± 0.12	0.43 ± 0.05
(gCOD_VFA_/(gCOD_X_·h))	0.51 ± 0.12	0.49 ± 0.13	0.51 ± 0.07
**qPHA** (Cmmol_PHA_/(Cmmol_X_·h))	0.33 ± 0.07	0.33 ± 0.10	0.34 ± 0.04
(gCOD_PHA_/(gCOD_X_·h))	0.40 ± 0.08	0.39 ± 0.11	0.39 ± 0.04
**PHA_max_feast_** (wt.% VSS basis)	21.6 ± 1.9	22.4 ± 5.0	24.2 ± 3.8
**Y_PHA/VFA_** (Cmmol_PHA_/Cmmol_VFA_)	0.81 ± 0.01	0.77 ± 0.03	0.79 ± 0.04
(gCOD_PHA_/gCOD_VFA_)	0.78 ± 0.06	0.78 ± 0.04	0.76 ± 0.04
**Y_X/VFA_** (Cmmol_X_/Cmmol_VFA_)	0.08 ± 0.01	0.05 ± 0.01	0.04 ± 0.01
(gCOD_X_/gCOD_VFA_)	0.07 ± 0.01	0.05 ± 0.01	0.03 ± 0.01
**Y_X/PHA_** (Cmmol_X_/Cmmol_PHA_)	0.31 ± 0.06	0.27 ± 0.05	0.24 ± 0.05
(gCOD_X_/gCOD_PHA_)	0.25 ± 0.05	0.23 ± 0.04	0.21 ± 0.06
**HB:HV** (wt.%)	55:45	57:43	46:54

**Table 2:** Alpha diversity indexes.

Index	Day-0	Day-21	Day-49	Day-63
Simpson_1-D	0.9	0.9	0.9	0.7
Shannon_H	3.4	2.6	3.0	2.0
Fisher_alpha	249.4	111.0	113.5	112.5
Chao-1	82.0	71.0	71.0	71.0

## Data Availability

Not applicable.
